# Incidence and risk factors of hospitalization for bronchiolitis in preterm children: a retrospective longitudinal study in Italy

**DOI:** 10.1186/1471-2431-9-56

**Published:** 2009-09-10

**Authors:** Patrizio Pezzotti, Jessica Mantovani, Nicoletta Benincori, Eleonora Mucchino, Domenico Di Lallo

**Affiliations:** 1 Agency for Public Health of Lazio region, Scientific Direction unit, Rome, Italy; 2 Agency for Public Health of Lazio region, Health Protection department, Rome, Italy; 3 Giovanbattista Grassi Hospital, Neonatology and Paediatric unit, Rome, Italy; 4 San Giuseppe Hospital, Neonatology and Paediatric unit, Marino (Rome), Italy

## Abstract

**Background:**

Bronchiolitis is a distressing, potentially life-threatening respiratory condition that affects infants. We evaluated the incidence and risk factors of hospitalization for broncholitis in preterm infants (i.e., a gestational age of <36 weeks) born between 2000 and 2006, and the use and impact of Palivizumab, a monoclonal antibody that in randomized clinical trials has been shown to lessen the severity of RSV-related bronchiolitis.

**Methods:**

Retrospective cohort study that linked data from four health administrative databases in the Lazio region (a region of central Italy): the birth register, the hospital discharge register, and two ad-hoc databases that record the doses of Palivizumab administered at two local health units.

**Results:**

Among 2407 preterm infants, 137 had at least one hospitalization for bronchiolitis in the first 18 months of life, an overall incidence rate of 4.70 per 100 person-years (95%CI: 3.98-5.56); similar incidence rates were observed by calendar year. A multiple Poisson model showed that the following characteristics were associated with higher incidence: younger age of the infant, the period between October-April, male gender, low Apgar score at birth, low birth weight, and low maternal educational level. At least one dose of Palivizumab was administered to 324 (13.5%) children; a dramatic increase from 2000 (2.8%) to 2006 (19.1%) (p < 0.01) was observed. Other factors independently associated with more frequent Palivizumab use were older maternal age, Italian-born mothers, female gender, low Apgar score, low birth weight, shorter gestational age, a diagnosis of broncho-dysplasia, and the month of birth. It is of note that none of the 34 children with congenital heart disease were prescribed Palivizumab. Performing several multiple Poisson models that also considered Palivizumab use as covariate, although the point estimates were in agreement with previous clinical trial results, we did not find in most of them a significant reduction for immunized children to be hospitalized for bronchiolitis.

**Conclusion:**

In Italy the incidence of hospitalization for bronchiolitis, and its associated risk factors, are similar to that found in other countries. Although Palivizumab use is associated with the most important characteristics of severe prematurity, other aspects of its non-use in children with congenital heart disease, the age and the birth country of the mother suggest the need for public health measures that can reduce these health disparities. Finally, the estimated effectiveness of Palivizumab in routine practice, although not significant, confirms the results of previous clinical trials, but its impact on modifying the temporal trend in this population is still negligible.

## Background

Bronchiolitis is an acute respiratory illness that particularly affects infants and young children characterized by coryza and sometimes low-grade fever that progress over a few days to cough, tachypnoea, hyperinflation, chest retraction, and widespread crackles, wheezes, or both [1].

The incidence of bronchiolitis has a seasonal trend with a peak during the winter months in temperate climates, during the cool rainy season in tropical and subtropical areas, and during the cool dry season in South America and South Africa [2]. A study in the US in the mid-nineties estimated that around 10% of children have bronchiolitis in the first year of life [3]. However, this estimate for Canada in 2003 was around 4% [4].

Hospital admission rates for bronchiolitis in the US and Europe are reported to be around 3% for children less than 1 year of age [2]. Independent factors typically associated with a high risk of hospitalization are male gender, premature birth without or, especially, with broncho-displasya, congenital heart disease, T-cell immunodeficiency, age <6 months, birth during the first half of the influenza season, and crowding/siblings [5,6].

It has been estimated that the respiratory syncytial virus (RSV) is the etiologic agent in more than 70% of the cases (80%-90% during the winter) [7]. A study in the mid-nineties evaluated the safety and efficacy of Palivizumab, a humanized murine monoclonal anti-F glycoprotein antibody preparation, in preterm infants with and in those without chronic lung disease. The study showed the preparation to be safe and efficacious in reducing RSV-associated hospitalizations by 55% [8]. A subsequent study conducted between 1998-2002 in infants ≤ 24 months of age with documented hemodynamically significant congenital heart disease (CHD) showed a 45% relative reduction in RSV-associated hospitalizations [9].

Palivizumab was introduced in Italy in 1999 and it is administered following international and national guidelines [10-12]: at-risk children are identified by the neonatologists in the perinatal units, their families are then contacted and offered the prophylaxis free of charge. The proposed schedules repeat administrations every 30 days during the epidemic period.

Until now, several studies have evaluated the impact of introducing Palivizumab prophylaxis on the incidence of hospitalization with bronchiolitis [13-17] but none of these have been conducted in Italy. The objectives of this study were thus to evaluate the incidence rate and factors associated with preterm infant hospitalizations for bronchiolitis, and the use and the impact of Palivizumab in this country.

### Methods

#### Study design, setting, participants, and data sources

This is a retrospective longitudinal study of preterm infants (i.e., <36 weeks of gestational age) born between 2000-2006 whose mothers at time of delivery were living in the catchment area of two local health units (LHUs) in the Lazio region, central Italy. Both LHUs are located in the county of Rome, one in the city center and the other in a suburban area. During the study period the population in these two LHUs slightly increased from more than 985,000 at the beginning of 2000 to more than 1,055,000 people at the end of 2006. In this period 67,292 children were born to resident mothers identified using the birth register ("Certificati di assistenza al parto" in Italian, hereafter called CEDAP). Details about CEDAP can be found in previous publications [18,19]. Briefly, this register reports information on socio-demographic characteristics of both parents (age, education, occupational status, etc.), obstetric history and pregnancy (previous pregnancies and/or abortions, duration, characteristics, etc.) and prenatal care (clinical examinations, ultrasounds, amniocentesis, etc.), delivery (place, type, etc.), and information on the newborn (gender, birth order, birth weight, gestational age, Apgar score at 5 minutes, etc.). Hospital admissions for bronchiolitis were identified in the hospital discharge database of the Lazio region ("Sistema Informativo Ospedaliero della Regione Lazio", hereafter called SIO). Details about the SIO can be found in previous publications [20,21]. Briefly, this database was started in 1994 and all hospitals in the region are required to record data on a standardized form of admission and discharge dates, personal data of the patient (i.e., date of birth, gender, name, surname, municipality of residence, nationality), the principal diagnosis and up to five secondary diagnoses [coded by the International Classification of Diseases - ninth revision (ICD-9)], diagnostic procedures (also coded by the ICD-9), and death, if it occurred during the hospitalization. Hospitalizations for bronchiolitis were identified by the ICD-9 code 466.11 or 466.19, reported either as the first or secondary diagnosis. Although only code 466.11 refers to bronchiolitis due to VRS, we also included codes for "other" or "unknown" etiologies. It is of note that in clinical practice the etiology of bronchiolitis is very often not determined because it does not change the course of treatment in infants. Only subjects hospitalized for bronchiolitis before age three were included. The SIO was also used to identify infants with a diagnosis of broncho-dysplasia and CHD. It is of note that it is extremely unlikely that a hospitalization for bronchiolitis would be missed because SIO covers the entire region, not only the territory of the LHUs involved.

Data about each single dose of Palivizumab were collected in ad-hoc databases that the two LHUs created for administrative purposes. In accordance with the national guidelines the prophylaxis was strongly recommended for children with a gestational age of <32 weeks and aged <1 year at the beginning of the epidemic period. The prophylaxis is also strongly recommended in children with chronic lung disease and in those with hemodynamically congenital heart disease (excluding those who had a surgical and radical correction and without need of pharmacological treatment) and aged <2 years at the beginning of the epidemic period. The prophylaxis was also recommended for infants born between 32 and 35 weeks, for those aged <1 year at the beginning of the epidemic period and for those with at least two more known risk factors associated with the disease (e.g., exposure to passive smoking, not breastfed). If the child was hospitalized, the prophylaxis was offered at the hospital. After discharge, the public pediatric ambulatory, warned by the clinicians, contacts the family if prophylaxis is indicated for the infant. Prophylaxis was usually repeated once a month during the epidemic period.

### Cross linkage of the data sources

Data from the various sources were initially linked using the complete surname, name, gender and date of birth of the child. This link was performed using the SAS program (version 8.2). For subjects not linked using those criteria, the SALI (software for automated linkage in Italy) program was used to match individual records. SALI reduces false negatives by taking into account possible errors in key fields [22]. Finally, for immunized children and for children hospitalized for bronchiolitis not linked with CEDAP after the previously described automated cross-linkages, the CEDAP was checked manually for all children born on the date of birth of any immunized or hospitalized child unidentified in the previous steps.

### Statistical analysis

The analysis initially included 2558 children born between 2000-2006 in the two LHUs for whom the reported gestational age in the CEDAP was <36 weeks. We then excluded 151 of them whose date of discharge after delivery was after 31/12/2006 or not listed, or the gender was not reported, or who died immediately after birth.

The outcome of the analysis was initially any hospitalization for bronchiolitis within the first three years of age. However, the analysis we conducted was restricted to first hospitalizations within the first 18 months, because only six cases had more than one hospitalization for bronchiolitis and only two cases were hospitalized after 18 months of age. The incidence rate was calculated as the ratio of the hospitalizations for bronchiolitis within 18 months of age out of the person-time at risk. For each child, the person-time at risk was calculated from the date of discharge after delivery until the first hospitalization for bronchiolitis, the date the child turned 18 months of age, and the first of January 2007. Incidence rates of hospitalization for bronchiolitis were also calculated stratifying for several of the infants' characteristics at birth [i.e., birth weight (<1000, 1000-2000, >2000 grams), gestational age (<32, 32-36 weeks), gender, year of birth, Apgar score at 5 minutes after birth (≤ 7, >7), reported diagnosis of broncho-dysplasia during the delivery hospitalization or in subsequent hospitalizations (ICD9-CM code: 770.7 in any diagnostic position], reported diagnosis of CHD at delivery or at subsequent hospitalizations (ICD9-CM codes: 745.0, 7451.10, 745.11, 745.2, 745.3, 745.69, 746.01, 746.02, 746.1, 746.2, 746.7, 746.89, 747.22, 747.3 in any diagnostic position)] and of maternal characteristics [i.e, age at delivery (i.e.,≤ 32, >32 years, where 32 is the median age), years of education (≤ 8 years, >8 years, unknown), country of birth (Italy, other), and parity (0,1, ≥ 2)]. Incidence rates were also calculated stratifying for age of the infants (i.e. in months and into three groups: <6, 6-11, 12-18 months) and calendar month. Crude and adjusted incidence rate ratios (IRR) of bronchiolitis hospitalizations for the previously described characteristics were estimated using univariate and multiple Poisson models.

Descriptive frequency tables of the previously described characteristics and use of Palivizumab were provided. To evaluate the association of some characteristics with Palivizumab use we calculated the Chi-squared test. To evaluate the independent association of these characteristics with the administration of Palivizumab, a multiple logistic analysis was performed.

**Figure 1:**
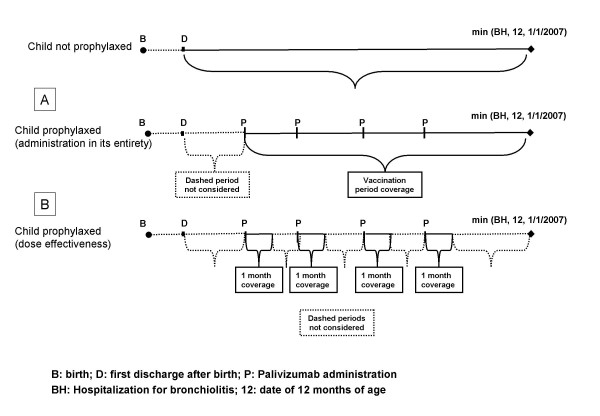
**Graphical representation of how person-time at risk of being hospitalized for bronchiolitis was calculated for non-prophylaxed and prophylaxed infants**. For prophylaxed infants, two different approaches were proposed. Box A shows that person time was calculated since the first date of Palivizumab administration to the first hospitalization for bronchiolitis, or the date the subject turned 18 months of age, or the first of January 2007, whichever comes first. Box B shows that, compared to the previous approach, the person-time does not include the time-periods exceeding the 30 days after each Palivizumab administration. In this case also hospitalizations for bronchiolitis happened in the dashed periods are not considered.

To evaluate the impact of the administration of Palivizumab on the risk of being hospitalized, we performed additional Poisson models that estimated IRRs for immunized compared to non-immunized infants using two different approaches of selecting specific at risk time periods among those who received prophylaxis. The first approach evaluated the effectiveness of the immunization in its entirety considering as the at-risk-time the period since the date of the first dose of Palivizumab administered to the first hospitalization for bronchiolitis, or the date the subject turned 18 months of age, or the first of January 2007, whichever comes first (see section A of Figure 1). This approach evaluated the effect of the immunization strategy in its entirety and hereafter is referred to as the vaccination-strategy effectiveness. The second approach evaluated the specific effect of Palivizumab in the 30 days after each dose. This means that all time periods and events 30 days after each dose was administered were not considered in the analysis. This approach, hereafter referred to as the dose-effectiveness approach, mimics the proposed scheme of administration and it evaluates the "absolute" effect of the prophylaxis only in the 30 days following each administration (see section B of Figure 1). For both approaches a multiple Poisson model, including the previously described characteristics as possible confounders, was performed. Furthermore, this model was also performed 1) restricting only to children born at <32 weeks gestation; 2) restricting the analysis to the time periods from the beginning of October to the end of April (i.e., the epidemic period) of each year. The analyses about the effectiveness of Palivizumab were restricted to the first year of life as this prophylaxis is not recommended after one year of age in most preterm infants without chronic lung disease.

### Study approval

Lazio Sanità-Agenzia di Sanità Pubblica is the governmental agency of the Lazio region responsible for health information systems (e.g., infectious disease notifications, hospital discharge records); the management of these data for public health purposes does not require a patient's informed consent nor it does require any authorization regarding privacy laws.

## Results

### Incidence and risk factors of hospitalization for bronchiolitis

During the period 2000-2006, 67,292 children were born to mothers living in the two LHUs of Rome; the median gestational age was 39 (mean = 38.9) weeks. Among them, 2407 (3.6%) were born at <36 weeks of gestation; the median gestational age among preterm infants was 34 (mean = 33) weeks and 201 (8.3% of preterm infants) were born at <29 weeks.

**Figure 2:**
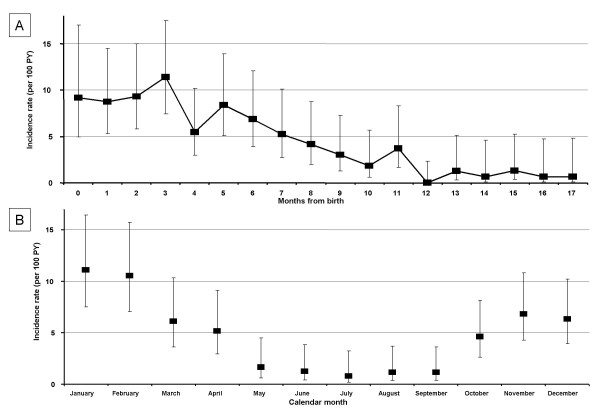
**Incidence rates of hospitalizations for bronchiolitis by age (in months) (section A) and by calendar month (section B) in pre-term infants; Rome, Italy 2000-2006**.

Table 1 shows the number of cases, the person-years and the incidence rates of a first hospitalization for bronchiolitis by age, by calendar month, and by calendar year among preterm infants. One hundred thirty-seven were hospitalized at least once with a diagnosis of bronchiolitis within the first 18 months of age. Overall, the incidence rate was 4.70 per 100 person-years (PY) (95%CI: 3.98-5.56). Significantly higher incidence rates were observed in the first six months of life and the incidence rates decreased with age (p < 0.01). This significantly decreasing trend was also observed by month of age (Figure 2, section A) (p < 0.01). Incidence rates also varied significantly by calendar month with the highest rates observed in the period October-April (hereafter defined as the epidemic period), with a peak in January (Figure 2, section B); the incidence rate was 7.17 and 1.22 per 100 PY in the epidemic and in the non-epidemic period (p < 0.01), respectively. Table 2 shows the incidence rates of a first hospitalization for bronchiolitis stratified for several characteristics. Significant higher incidence rates were observed for children born to mothers with ≤ 8 years of education compared to those with >8 years (p = 0.02). Characteristics of the infants that were significantly associated with higher incidence rates included: male gender (p = 0.02), low birth weight (p < 0.01), gestational age <32 weeks (p = 0.01), Apgar score ≤ 7, and presence of bronchodysplasia (p = 0.04); there was no statistical significance for year of birth (p = 0.61).

**Table 1:** Incidence rates of hospitalization for bronchiolitis in the first 18 months of age in premature infants, Rome, Italy, 2000-2006

	**Person years** **(PY)**	**Cases**	**Rate** **(per 100 PY)**	**95% CI**
**Age (months)**				
**[0 -- 6]**	986	88	8.93	7.24 -11.00
**(6 - 12]**	1005	42	4.18	3.09 -5.65
**(12 - 18]**	921	7	0.76	0.36 -1.59
				
**Calendar month**				
**January**	223	25	11.20	7.57 -16.57
**February**	226	24	10.64	7.13 -15.87
**March**	227	14	6.18	3.66 -10.43
**April**	229	12	5.23	2.97 -9.21
**May**	235	4	1.70	0.64 -4.54
**June**	238	3	1.26	0.41 -3.90
**July**	245	2	0.82	0.20 -3.27
**August**	251	3	1.20	0.39 -3.71
**September**	254	3	1.18	0.38 -3.66
**October**	257	12	4.67	2.65 -8.22
**November**	262	18	6.88	4.33 -10.91
**December**	266	17	6.39	3.98 -10.29
				
**Year of observation**				
**2000**	138	7	5.07	2.42 -10.63
**2001**	399	17	4.26	2.65 -6.85
**2002**	427	22	5.15	3.39 -7.82
**2003**	436	30	6.88	4.81 -9.84
**2004**	503	18	3.58	2.25 -5.68
**2005**	527	23	4.36	2.90 -6.56
**2006**	481	20	4.16	2.68 -6.44

**Total**	2912	137	4.70	3.98 -5.56

**Table 2:** Incidence rates of first hospitalization for bronchiolitis in the first 18 months of age in premature infants, stratified for several characteristics, Rome, Italy, 2000-2006

		**N**	**Person** **Years (PY)**	**Cases**	**Rate** **(per 100 PY)**	**95% CI**
**Age of the mother (years)**	≤ *32*	1119	1359.4	68	5.00	3.94 -6.34
	>*32*	1288	1553.1	69	4.44	3.51 -5.63
**Parity**	*0*	1457	1773.2	77	4.34	3.47 -5.43
	*1*	679	807.77	45	5.57	4.16 -7.46
	≥ *2*	271	331.55	15	4.52	2.73 -7.50
**Years of education**	≤ *8*	1085	1312.5	80	6.10	4.90 -7.59
	>*8*	1318	1597	56	3.51	2.70 -4.56
	*Unknown*	4	3	1	33.33	4.70 -236.64
**Birth-country of the mother**	*Italy*	2049	2505	119	4.75	3.97 -5.68
	*Other*	358	407	18	4.42	2.79 -7.02
**Gender**	*Males*	1282	1535.2	85	5.54	4.48 -6.85
	*Females*	1125	1377.3	52	3.78	2.88 -4.95
**Birth weight (grams)**	<*1000*	152	161.96	15	9.26	5.58 -15.36
	*1000-2000*	786	911.71	60	6.58	5.11 -8.48
	>*2000*	1469	1838.8	62	3.37	2.63 -4.32
**Gestational age**	<*32*	516	585.24	34	5.81	4.15 -8.13
	*32-35*	1891	2327.3	103	4.43	3.65 -5.37
**Apgar score**	≤ *7*	395	456	36	6.57	4.74 -9.11
	>*7*	2012	2456	101	3.43	2.82 -4.17
**Broncho dysplasia**	*No*	2346	2850	131	4.60	3.87 -5.46
	*Yes*	61	63	6	9.54	4.29 -21.24
**Congenital heart disease**	*No*	2373	2873	134	4.66	3.94 -5.52
	*Yes*	34	40	3	7.58	2.44 -23.50

Table 3 shows the crude (CIRR) and adjusted incidence rate ratios (AIRR) of being hospitalized for bronchiolitis. After adjusting for all characteristics, statistically significant higher risks were still found for children born to mothers with ≤ 8 years of education, for males, in the first months of age, for low birth weight infants, for children with an Apgar score ≤ 7, and in the epidemic period. Similar results were found performing other Poisson models where birth weight and gestational age were entered as categorical instead of continuous variables (data not shown in table).

**Table 3:** Crude (CIRR) and adjusted (AIRR) incidence rate ratios of hospitalization for bronchiolitis, Rome, Italy 2000-2006

		**CIRR (95% CI)**	**p-value**	**AIRR (95% CI)**	**p-value**
**Age of the mother (years)**	≥ *32 vs. <32*	1.13 (0.80 -1.58)	0.49	1.08 (0.75 -1.55)	0.67
**Parity**	*1 vs. 0*	1.28 (0.88 -1.86)	0.19	1.34 (0.91 -1.98)	0.14
	≥ *2 vs. 0*	1.04 (0.59 -1.83)	0.89	1.09 (0.60 -1.95)	0.78
**Years of education**	≤ *8 vs. >8*	1.74 (1.23 -2.46)	<0.01	1.71 (1.18 -2.48)	0.01
	*Not known vs. >8*	9.51 (0.88 -102.1)	0.06	9.82 (0.79 -122.4)	0.08
**Birth country of the mother**	*Italy vs. other*	1.07 (0.65 -1.78)	0.78	1.30 (0.73 -2.30)	0.37
**Gender **	*male vs female*	1.47 (1.03 -2.08)	0.03	1.48 (1.04 -2.10)	0.03
**Calendar year (ref. 2001)**	2000	1.19 (0.49 -2.86)	0.70	0.79 (0.33 -1.88)	0.60
	2002	1.21 (0.64 -2.28)	0.56	1.28 (0.68 -2.43)	0.44
	2003	1.61 (0.89 -2.93)	0.12	1.73 (0.95 -3.16)	0.07
	2004	0.84 (0.43 -1.63)	0.61	0.92 (0.47 -1.79)	0.81
	2005	1.02 (0.54 -1.92)	0.94	1.24 (0.66 -2.34)	0.50
	2006	0.98 (0.51 -1.87)	0.94	1.16 (0.60 -2.24)	0.66
**Age (months)**	*<6 vs. ≥ 12*	11.75 (5.44 -25.35)	<0.01	14.54 (6.75 -31.35)	<0.01
	*6-11 vs. ≥ 12*	5.50 (2.47 -12.24)	<0.01	5.98 (2.68 -13.35)	<0.01
**Epidemic period**	*Yes vs. No*	5.89 (3.44 -10.06)	<0.01	5.48 (3.22 -9.35)	<0.01
**Birth weight **	*per 100 g decrease*	1.05 (1.02 -1.07)	<0.01	1.06 (1.02 -1.11)	0.01
**Gestational age**	*per 1 week less*	1.08 (1.02 -1.14)	0.01	0.97 (0.88 -1.07)	0.58
**Apgar score**	≤ *7 vs. > 7*	2.06 (1.40 -3.03)	<0.01	1.57 (0.99 -2.48)	0.05
**Broncho-dysplasia**	*Yes vs. No*	2.08 (0.91 -4.75)	0.08	1.70 (0.68 -4.28)	0.26
**Congenital heart disease**	*Yes vs. No*	1.63 (0.51 -5.18)	0.41	1.64 (0.52 -5.19)	0.40

### Use of Palivizumab and estimates of its effectiveness on the risk of hospitalization for bronchiolitis

Overall, 324 (13.5%) children received at least one dose of Palivizumab. There were 1291 doses administered with a median of 4 doses received per child. Doses were administered exclusively between October and April, with the mode of administration in December (24.7% of the total). Table 4 shows maternal and infant characteristics stratified for Palivizumab use (i.e., no administration and at least one dose). During the study period, the percentage of children who received Palivizumab dramatically increased from 2.8% in 2000 to 19.1% in 2006. Palivizumab was more frequently administered to children born to mothers over 32 years of age, who had no other children, more than 8 years of education, and who were born in Italy; regarding the characteristics of the infants; Palivizumab was more frequently administered to females, with a birth weight of <1000 grams, who were born at <32 weeks, with an Apgar score of ≤ 7, with a diagnosis of bronchodysplasia, and to those born between July and December. It is of note that Palivizumab was not administered to infants with CHD. Among them, only 5 (14.7%) were born at <32 weeks of gestation, while 8 (23.5%) and 15 (44.1%) were born at 34 and 35 weeks, respectively. Multiple logistic regression showed statistically significant adjusted odds-ratio (AOR) >1 of receiving Palivizumab for preterm infants born to mothers >32 years old, for those born to Italian mothers, for females, for those born most recently, for those who weighed <2000 grams, for those born at <32 weeks, for those with an Apgar score ≤ 7, for those not born in the spring, and for those with a diagnosis of bronchodysplasia.

**Table 4:** Use of Palivizumab in preterm infants and adjusted odds ratios (AOR) by several characteristics; Rome, Italy 2000-2006

		***Yes***		**p value**	**AOR**	**(95%CI)**	**p** **value**
		***N***	**%**				

**Age of the mother (years)**	≤ *32 (REF)*	118	10.5	<0.01	1.00		<0.01
	>32	206	16.0		1.67	(1.22 -2.30)	
**Parity**	*0 (REF)*	221	15.2	<0.01		-	
	1	72	10.6		0.83	(0.58 -1.18)	0.30
	>1	31	11.4		0.92	(0.55 -1.54)	0.74
**Years of education of the mother**	≤ *8 years (REF)*	137	12.6	0.20	1.00	-	
	*>8 years*	186	14.1		1.14	(0.84 -1.54)	0.42
	*Not known*	1	25.0		NE	-	
**Country of birth of the mother**	*Italy (REF)*	283	13.8	0.23	1.00	-	<0.01
	*Other*	41	11.4		0.47	(0.30 0.73)	
**Gender**	*Male (REF)*	160	12.5	0.13	1.00	-	
	*Female*	164	14.6		1.34	(1.00 1.80)	0.05
**Year of birth**	*2000 (REF)*	9	2.8	<0.01	1.00	-	
	2001	22	7.0		2.48	(1.02 -6.00)	0.04
	2002	41	13.0		7.00	(3.05 -16.02)	<0.01
	2003	47	13.0		9.37	(4.13 -21.28)	<0.01
	2004	73	18.4		13.88	(6.23 -30.93)	<0.01
	2005	61	18.3		17.90	(7.87 -40.67)	<0.01
	2006	71	19.1		18.43	(8.19 -41.46)	<0.01
**Birth weight (grams)**	*<1000 (REF)*	76	50.0	<0.01	1.00	-	
	*1000-2000*	209	26.6		1.22	(0.75 -1.99)	0.42
	>2000	39	2.6		0.18	(0.10 -0.34	<0.01
**Gestational age (weeks)**	*<32 (REF)*	221	42.8	<0.01	1.00	-	
	32-35	103	5.4		0.19	(0.13 -0.27)	<0.01
**Bronchodysplasia**	*Yes (REF)*	41	67.2	<0.01	5.96	(2.90 -12.26)	<0.01
	*No*	283	12.1			-	
**Apgar score**	≤ *7*	128	32.4	<0.01	1.55	(1.10 -2.19)	0.01
	*>7 (REF)*	196	9.7			-	
**Month of birth**	*Jan-Mar*	71	12.6	<0.01	1.57	(1.00 -2.45)	0.05
	*Apr-Jun (REF)*	68	11.4		1.00	-	
	*Jul-Sep*	99	15.3		2.00	(1.32 -3.03)	<0.01
	*Oct-Dec*	86	14.4		2.12	(1.38 -3.26)	<0.01
**Congenital heart disease (CHD)**	*Yes (REF)*	0	0.0	0.02	1.00	-	
	*No*	324	13.6		NE	-	
							

**Total**		324	13.5				

AOR: Adjusted odds ratio of administration of Palivizumab; REF: reference group; NE: not estimable

Among the 324 preterm children who received at least one dose of Palivizumab, 8 (2.5%) were hospitalized with bronchiolitis in the first 12 months of life. Among them, 6 were hospitalized in the 30-day period just after each administration of Palivizumab. The incidence rate was 5.72 (95%CI: 2.86-11.44; PY = 140) per 100 PY, slightly lower than that observed in non-immunized children (6.97 per 100 PY, 95%CI: 5.84-8.32; PY = 1751). The incidence rate in the 30 days immediately after each administration of Palivizumab was 9.55 (95%CI: 4.29-21.27; PY = 63) per 100 PY. Table 5 shows the estimated crude and adjusted IRRs of hospitalization for bronchiolitis for those who received Palivizumab compared to those who did not, estimated by several Poisson models and with the two different approaches (see methods and figure 1). When adjusting for potential confounders, no significant risk reductions were found in th eproposed models, but one (i.e., Model 2, vaccination-strategy).

**Table 5:** Incidence rate ratios (IRR) of hospitalization for bronchiolitis in preterm infants immunized compared to those not immunized with Palivizumab, Rome, Italy 2000-2006

	**Vaccination-dose effectiveness**			**Vaccination-strategy effectiveness**		
	**IRR**	**95% CI**	**p-value**	**IRR**	**95% CI**	**p-value**

**Model 1: univariate analysis**	1,37	0,60 -3,11	0,45	0,82	0,40 -1,69	0,59
Model 2: multiple analysis*	0,51	0,22 -1,17	0,11	0,47	0,23 -0,98	0,04
**Model 3: as model 2 but restricted to GA<32 weeks **	0,67	0,24 -1,85	0,44	0,65	0,26 -1,63	0,36
**Model 4: as model 2 but restricted only to the epidemic period**	0,50	0,22 -1,14	0,10	0,49	0,24 -1,03	0,06

*adjusted for all covariates of table 3; GA = Gestational age

## Discussion

We evaluated the incidence and the risk factors of hospitalization for bronchiolitis in preterm children in and around Rome, Italy. Overall, the incidence of hospitalization was 4.70 per 100-PY in the first 18 months of age and this estimate is in agreement with that reported in other studies [6,16,23,24]. We found that the incidence strongly declined with age and that hospitalization after 18 months of age is extremely rare. Furthermore, we observed a strong seasonal effect with the highest monthly incidence estimated from October to April. Other independent significant risk factors of being hospitalized for bronchiolitis were: low Apgar score 5 minutes after birth, low birth weight, male gender, and low educational level of the mother (i.e., ≤ 8 years). While our results for some of these factors (i.e., young age, autumn/winter period, low birth weight, low Apgar score, and male gender) are in agreement with previous publications [2,5,6], low educational level has never before been identified as an independent risk factor for hospitalization for bronchiolitis. This could be a proxy of other factors, such as maternal smoking that was not evaluated in our study. It is of note that other factors, such as increasing birth order, a diagnosis of bronchodysplasia, CHD, and low gestational age, after having adjusted for the other covariates, were not significantly associated with being hospitalized for bronchiolitis, and this contrasts with previous publications [2]. The results regarding gestational age can be explained by their strong correlation with low birth weight; regarding increasing birth order, bronchodysplasia, and CHD, the estimated adjusted IRR still suggest that children with these characteristics had a greater risk of being hospitalized with bronchiolitis, and the fact that they were not found to be statistically significant is likely only the result of the limited statistical power due to the low numbers in these groups.

During the 2000-2006 study period we observed a significantly increasing trend of the percentage of pre-term children who received at least one dose of Palivizumab. After adjusting for other covariates, year of birth was still strongly associated with the administration of Palivizumab; other factors independently associated were low birth weight, shorter gestation, low Apgar score, reported diagnosis of bronchodysplasia, female gender, birth country and maternal age. While the first four factors were expected to be associated with Palivizumab prophylaxis, there is no clear explanation for the latter three. In particular, it was expected that male children, being at higher risk of hospitalization, were more likely to have received prophylaxis with Palivizumab than females and not vice versa. One possible explanation could be related to unknown, uncontrolled confounders for which the effect was indirectly expressed through gender [25]. Regarding maternal age and maternal birth country, the lower adjusted odds of receiving at least one dose of Palivizumab could reflect, as a proxy, lower social economic status. Furthermore, for mothers born outside Italy there could have been difficulties for the LHUs in contacting or in communicating with them.

We evaluated if there was a risk reduction of hospitalization for bronchiolitis in infants who received at least one dose of Palivizumab with two different analyses trying to identify both the effectiveness of each single dose and of the scheduled immunization program offered. In both cases we did not find, using different multiple models and different selected populations, a signficant risk reduction, but in one model (Model 2, Vaccination strategy in Table 5). However, all proposed models have point estimates that are similar ranging between 33% and 53% in Table 5. Although our estimates of the risk reduction were a little bit lower than the two clinical trials performed [8,9], we cannot exclude that this different magnitude of the effect simply resulted from chance. Furthermore, it is of note that the all hospitalizations studied in the two clinical trials had RSV antigen positive tests while we studied any hospitalization for bronchiolitis independent of RSV test results. As we specified in the methods section, the identification of the etiological agent of bronchiolitis in clinical practice is not always performed because it does not change the type of treatment given. Assuming that RSV-related hospitalizations for bronchiolitis were, as reported in the literature [2], 70% of the total, and that Palivizumab does not have any effect on non-RSV-related cases of bronchiolitis, we can further reduce our estimates of the effectiveness of Palivizumab on bronchiolitis hospitalizations of 30%. This implies that our risk reduction estimates of hospitalizations for RSV-related bronchiolitis should range between 53% and 67%, very similar to those previously reported in the two clinical trials.

It is of note that our data did not show any impact on the trend of the hospitalizations for bronchiolitis as shown by the incidence rates and the adjusted IRR by calendar year (see table 2 and 3). Even though the use of Palivizumab dramatically increased between 2000 and 2006, less than 20% of the preterm children born between 2004 and 2006 received the prophylaxis and the expected number of cases that were prevented is very low.

Before drawing conclusions we should consider some limitations of this study. First, this study was based on data collected only by two LHUs and results are limited by the small study size. Second, this was a retrospective observational study and the measured characteristics for children at birth, the date of Palivizumab administration, and the hospitalizations were taken from large databases created for other purposes. Third, we identified bronchiolitis hospitalizations using the ICD9-CM diagnostic codes 466.11 or 466.19. It is of note that in many cases the code 466.19 (bronchiolitis associated to a non-RSV or unknown infectious agent) was reported in the hospital discharge form; this most likely occurred because no diagnostic exams had been performed to identify the underlying infectious agent. This did not permit us to perform specific analyses of RSV-associated bronchiolitis. However, many studies reported that between 70% and 90% of hospitalizations for bronchiolitis in infants are RSV-associated (2). Finally, preterm infants have a high risk of death and we cannot exclude that for some of them we could have overestimated the person-time at risk of broncholitis if they died before 18 months of age. However, since we excluded those who died during their first hospital admission the impact of this issue on our results should be limited.

## Conclusion

In conclusion, this is the first report since the introduction of Palivizumab to evaluate the incidence and risk factors associated with hospitalizations for bronchiolitis in preterm infants in Italy. We highlighted that in Italy the incidence of hospitalization for bronchiolitis and the risk factors associated with it are similar to those found in other countries. Although the use of Palivizumab is associated with the most important characteristics of severe prematurity, its non-use in children with congenital heart disease contrasts with international guidelines; furthermore, its association with maternal age and the country of birth suggests the need for public health measures that can reduce health disparities.

## Competing interests

The authors declare that they have no competing interests.

## Authors' contributions

PP conceived the study design, supervised all the phases of the study, planned and performed the statistical analysis, drafted and revised the manuscript. JM contributed to the design of the study, performed the record linkages among the health information systems, performed the statistical analyses, drafted and revised the manuscript. NB and EM conceived of the initial idea of the study, collected all the data regarding Palivizumab administration in their own LHU, revised the manuscript. DDL conceived of the initial idea of the study, participated in the design of the design, revised the manuscript and contributed especially to the intellectual content. All authors read and approved the final manuscript.

## Pre-publication history

The pre-publication history for this paper can be accessed here:


